# Passive Sensor Data for Characterizing States of Increased Risk for Eating Disorder Behaviors in the Digital Phenotyping Arm of the Binge Eating Genetics Initiative: Protocol for an Observational Study

**DOI:** 10.2196/38294

**Published:** 2022-06-02

**Authors:** Robyn E Kilshaw, Colin Adamo, Jonathan E Butner, Pascal R Deboeck, Qinxin Shi, Cynthia M Bulik, Rachael E Flatt, Laura M Thornton, Stuart Argue, Jenna Tregarthen, Brian R W Baucom

**Affiliations:** 1 Department of Psychology University of Utah Salt Lake City, UT United States; 2 Department of Psychiatry University of North Carolina at Chapel Hill Chapel Hill, NC United States; 3 Department of Nutrition University of North Carolina at Chapel Hill Chapel Hill, NC United States; 4 Department of Medical Epidemiology and Biostatistics Karolinska Institutet Stockholm Sweden; 5 Department of Psychology and Neuroscience University of North Carolina at Chapel Hill Chapel Hill, NC United States; 6 Recovery Record San Francisco, CA United States

**Keywords:** digital phenotyping, eating disorders, personal digital devices, methodology

## Abstract

**Background:**

Data that can be easily, efficiently, and safely collected via cell phones and other digital devices have great potential for clinical application. Here, we focus on how these data could be used to refine and augment intervention strategies for binge eating disorder (BED) and bulimia nervosa (BN), conditions that lack highly efficacious, enduring, and accessible treatments. These data are easy to collect digitally but are highly complex and present unique methodological challenges that invite innovative solutions.

**Objective:**

We describe the digital phenotyping component of the Binge Eating Genetics Initiative, which uses personal digital device data to capture dynamic patterns of risk for binge and purge episodes. Characteristic data signatures will ultimately be used to develop personalized models of eating disorder pathologies and just-in-time interventions to reduce risk for related behaviors. Here, we focus on the methods used to prepare the data for analysis and discuss how these approaches can be generalized beyond the current application.

**Methods:**

The University of North Carolina Biomedical Institutional Review Board approved all study procedures. Participants who met diagnostic criteria for BED or BN provided real time assessments of eating behaviors and feelings through the Recovery Record app delivered on iPhones and the Apple Watches. Continuous passive measures of physiological activation (heart rate) and physical activity (step count) were collected from Apple Watches over 30 days. Data were cleaned to account for user and device recording errors, including duplicate entries and unreliable heart rate and step values. Across participants, the proportion of data points removed during cleaning ranged from <0.1% to 2.4%, depending on the data source. To prepare the data for multivariate time series analysis, we used a novel data handling approach to address variable measurement frequency across data sources and devices. This involved mapping heart rate, step count, feeling ratings, and eating disorder behaviors onto simultaneous minute-level time series that will enable the characterization of individual- and group-level regulatory dynamics preceding and following binge and purge episodes.

**Results:**

Data collection and cleaning are complete. Between August 2017 and May 2021, 1019 participants provided an average of 25 days of data yielding 3,419,937 heart rate values, 1,635,993 step counts, 8274 binge or purge events, and 85,200 feeling observations. Analysis will begin in spring 2022.

**Conclusions:**

We provide a detailed description of the methods used to collect, clean, and prepare personal digital device data from one component of a large, longitudinal eating disorder study. The results will identify digital signatures of increased risk for binge and purge events, which may ultimately be used to create digital interventions for BED and BN. Our goal is to contribute to increased transparency in the handling and analysis of personal digital device data.

**Trial Registration:**

ClinicalTrials.gov NCT04162574; https://clinicaltrials.gov/ct2/show/NCT04162574

**International Registered Report Identifier (IRRID):**

DERR1-10.2196/38294

## Introduction

### Background

The widespread adoption of smartphones and consumer wearables (eg, the Fitbit and Apple Watch) by the public provides clinical researchers with exciting opportunities for studying and augmenting interventions for mental health disorders. However, this innovation also presents unique complications when using personal digital devices for research purposes. To maximize the potential of these opportunities, it is necessary for mental health researchers to be aware of both the capabilities and limitations of personal digital devices. We provide a general introduction to these methodological issues and describe the use of personal digital devices for characterizing binge and purge episodes in the Binge Eating Genetics Initiative (BEGIN) [1].

Over the past decade, advances in digital technology have led to a significant increase in the affordability and accessibility of personal digital devices, including smartphones and consumer wearables. Current estimates suggest that approximately 80% of the global population are active smartphone users, which is up from the 49% reported in 2016 [2]. Furthermore, although smartphone ownership is highest among individuals 18 to 49 years old in countries with advanced economies [3], growing research suggests that the digital divide is also narrowing among older adults [4], individuals affected by serious mental illness [5], and in countries with emerging economies [3]. Therefore, by capitalizing on the near ubiquity and the acceptability of personal digital devices, mental health researchers have the opportunity to reach larger and more representative populations than ever before.

In addition to the opportunities for increased scalability in mental health research, advances in the capabilities of personal digital devices also provide researchers with unprecedented access to real time behavioral and physiological data. Especially valuable is the ability to use personal digital devices to access data that are collected unobtrusively and continuously while individuals go about their daily lives. These types of data are commonly referred to as “passive” data and include sensor data (eg, GPS location, locomotion, and light level) and higher order features derived from the sensors (eg, type of activity and sleep), as well as information about device usage (eg, call and text logs or social media usage), proximity to other personal digital device users via Bluetooth, voice samples, lexical analysis, and metadata (eg, battery level and time to respond to a text or questionnaire).

In combination with self-reported (or “active”) data collected from personal digital device users, researchers are able to use passive data to draw inferences about the individual and contextual factors preceding and following a particular behavior or event of interest. This process of using digital traces to draw inferences about individuals’ psychological state is a methodology commonly referred to as digital phenotyping. Similar to other terms, such as personal sensing [6,7], reality mining [8], and personal informatics [9], digital phenotyping was defined by Torous and colleagues [10] as the “moment-by-moment quantification of the individual-level human phenotype in situ using data from smartphones and other personal digital devices.” Digital phenotyping studies have gained increasing popularity in mental health research in the past 5 years and are being conducted on an ever-increasing range of psychological disorders (eg, social anxiety [11], depression and bipolar disorder [12], psychosis [13,14], and suicidal or nonsuicidal self-injury, the last for which Torous et al provide a review of the research [15]).

The seemingly limitless opportunities that personal digital devices offer mental health researchers are accompanied by several unique challenges, many of which stem from the fact that smartphones and consumer wearables are designed with user experience at the forefront. These devices must be aesthetically pleasing; easy to use, carry, or wear; have sufficient battery life and storage capacity; and provide their users with a high level of data privacy and security. However, achieving these goals comes at the expense of data that mental health researchers might desire. For example, to preserve battery power and optimize user experience, data collected from personal digital devices may be available at a less intensive sampling rate than what would be available from a comparable research grade device [16]. Similarly, to maximize user safety and privacy, data collected from personal digital devices are typically available in the form of summary values (eg, daily step count) created by proprietary algorithms. These design elements present challenges for researchers regarding what the data actually represent, and make it difficult to combine data collected from different devices or operating systems due to inconsistencies in the algorithms used. To address the issues of proprietary algorithms, multiple companies now exist (eg, Beiwe, the AWARE Framework, and The Effortless Assessment of Risk States [EARS] Tool) to provide researchers access to raw sensor data at the desired sampling rate as well as to higher order features (eg, activity and sleep) that can be collected consistently across devices and brands. These services, however, add a significant cost to researchers’ budgets, thereby decreasing some of the benefits implied by relying on participants’ existing personal digital devices. Finally, although the idiographic nature of personal digital device data presents several research opportunities, it also introduces many methodological challenges for handling missing data. Because each individual interacts with their personal digital device differently and because individual usage patterns change over time (eg, due to software updates or new applications and device features), it becomes increasingly challenging for researchers to make assumptions about the sources of missing data.

To illustrate these overarching issues in digital phenotyping research, we describe the challenges encountered and the methods used to address them in the BEGIN study—an ongoing study of BED and BN. The digital phenotyping arm of BEGIN is motivated by circumstances that are likely similar to many other studies using personal digital device data with a digital phenotyping approach. Though a number of interventions for BED and BN have at least some empirical support, between 20% and 65% of individuals who complete current best-available treatments demonstrate improvement; and, of those who do improve during treatment, only around half sustain those gains beyond 6 months after the end of treatment (this topic has been reviewed by Linardon et al [17], Peat et al [18], and Smink et al [19]). One particularly important treatment target in these interventions is the disordered eating behaviors that characterize BED and BN, which include binges (ie, episodes of uncontrollable eating), purges (ie, vomiting and misuse of laxatives or diuretics), and other compensatory behaviors (eg, excessive exercise and fasting). These behaviors have a clear beginning and end and happen repeatedly at varying time intervals both within and across individuals. An ideal intervention for disordered eating behaviors would therefore (1) help an individual recognize that they are at risk for engaging in one of these behaviors, (2) assist the individual in pre-empting the behavior, and (3) direct them toward coping strategies that are widely available and applicable to a large proportion of individuals with BED or BN (ie, cognitive, behavioral, or mindfulness interventions). The digital phenotyping arm of the BEGIN study is designed to accomplish the first step in the development of such an intervention by using sensor data from a consumer wearable to detect high-risk periods for a binge or purge episode.

### Objectives

In this protocol paper, we describe the digital phenotyping component of BEGIN, a multipronged research study that is examining the etiology of BED and BN and developing indicators of risk, course of illness, and treatment response in individuals with BED and BN using genetic, gut microbiota, and digital phenotypic data. The focus of this paper is on how we addressed the methodological challenges associated with using “out of the box” passive sensor data (ie, heart rate and step count) and active data (ie, feelings and behaviors) collected via iPhone and Apple Watch devices. Our objective is to provide a thorough and transparent description of the steps we took and the decisions we made when preparing these data for analysis.

## Methods

### Ethics Approval and Funding

All BEGIN study procedures were approved by the University of North Carolina Biomedical Institutional Review Board (17-0242). Funding for this study from the National Institute of Mental Health (NIMH) commenced on November 20, 2019. The study was registered on ClinicalTrials.gov (NCT04162574) on November 14, 2019. Funding from other sources, including the National Eating Disorders Association, Foundation of Hope, and Brain and Behavior Research Foundation, allowed us to start a feasibility pilot prior to receiving NIMH funding. Therefore, trial registration is considered to be retrospective.

### Study Design

Complete details about participant recruitment, study design, and procedures have previously been published [1]. In summary, participants were eligible if they were United States residents; existing iPhone users; between 18 and 45 years old; met the criteria of the Diagnostic and Statistical Manual of Mental Disorders, Fifth Edition for lifetime BED or BN; reported experiencing current binge eating episodes; and had not received bariatric surgery, recent inpatient treatment, or hospitalization for eating disorders. All participants provided digital informed consent through the Recovery Record app to have specific types of their activity and self-reported data harvested for the purposes of this study. Once enrolled, participants used the Recovery Record app to record real time assessments of feelings and eating behaviors on their personal iPhone and then on a first-generation Apple Watch, which they received in the mail approximately 7 days after study enrollment. A version of the Recovery Record app adapted for the Apple Watch was loaded onto all the watches. Participants were instructed to wear the Apple Watch consistently throughout the 30-day study period to track heart rate and actigraphy data (only removing it for bathing, sleeping, and recharging the battery). Thirty days after enrollment, Recovery Record stopped sharing all active and passive data with the BEGIN study and participants were able to keep the Apple Watch for personal use.

### Data Privacy and Confidentiality

To maximize data privacy and security, data from all sources were encoded and can only be matched using a key maintained in a password-protected file that can be accessed only by approved study personnel. Study data collected from the Recovery Record app and Apple Watches were maintained by Recovery Record and transferred with end-to-end encryption and authentication protocols. Further details have been described previously [1].

### Data Sources

Complete details for all BEGIN study data sources have been described previously [1]. A summary of the data sources relevant to the current protocol are presented below.

#### Participant Characteristics

Demographic data (age, sex, gender, ethnicity, and race) and lifetime eating disorder diagnostic history were collected from participants at the beginning of the study. The final sample included 96 participants from the feasibility pilot study. Pilot study participants were asked to report their biological sex (male or female), whereas participants in the main BEGIN study were asked to indicate which gender they most identified with (male, female, or other). Self-reported ethnic (Hispanic or non-Hispanic) and racial categories (African American or Black; Asian; American Indian or Alaska Native; Pacific Islander or Native Hawaiian; White; or other) provided to participants were consistent across the feasibility pilot and main study.

#### Active Data Collection

##### Daily Feelings and Meal Records

Participants were prompted 6 times per day, corresponding with 3 meals and 3 snack times, to complete meal records within the iPhone Recovery Record app. Meal records could not be logged on the Apple Watch. These meal records consisted of questions about meal characteristics (eg, what was eaten, how long ago, with whom, and where), current feelings (measured on a visual analogue scale from 0, depressed, to 100, overjoyed), and eating behaviors (eg, meal skipping). Participants also had the option to record additional information, including whether they engaged in or had the urge to engage in disordered eating behaviors (eg, binge eating or purging).

##### Disordered Eating Records

To capture event-contingent intensive measurements of disordered eating behaviors, participants were instructed to record all binge and purge episodes in the Apple Watch Recovery Record app. Within the app, participants indicated which behavior they engaged in and when it happened, with response options ranging from “right now” to “30 minutes ago” in 5-minute increments. Participants were additionally asked to record the strength of any urges to engage in disordered eating behaviors on a 5-point scale, ranging from “not at all” to “overbearing,” as well as to indicate their current feelings on an intensity scale of 0 to 100. Although participants were instructed to record binge or purge episodes, urges, and feeling ratings in the Apple Watch Recovery Record app, they also had the capability to do so in the iPhone version of the app. On the iPhone version of the app, participants could enter the exact time of a binge or purge event, meaning that on the iPhone, it was possible to record an event that happened more than 30 minutes previously.

#### Continuous Passive Data Collection: Heart Rate and Step Records

Heart rate data were collected passively from participants with the Apple Watch and harvested by the Recovery Record app using Apple’s native application programming interface. When the Apple Watch is charged and worn on the wrist, its sensor turns on approximately every 5 minutes to record heart rate at a frequency of 100 Hz using photo plethysmography. The number and timing of steps was also collected using the Apple Watch’s application programming interface and the Recovery Record app.

### Data Analytic Plan

#### Data Cleaning

**Figure 1:**
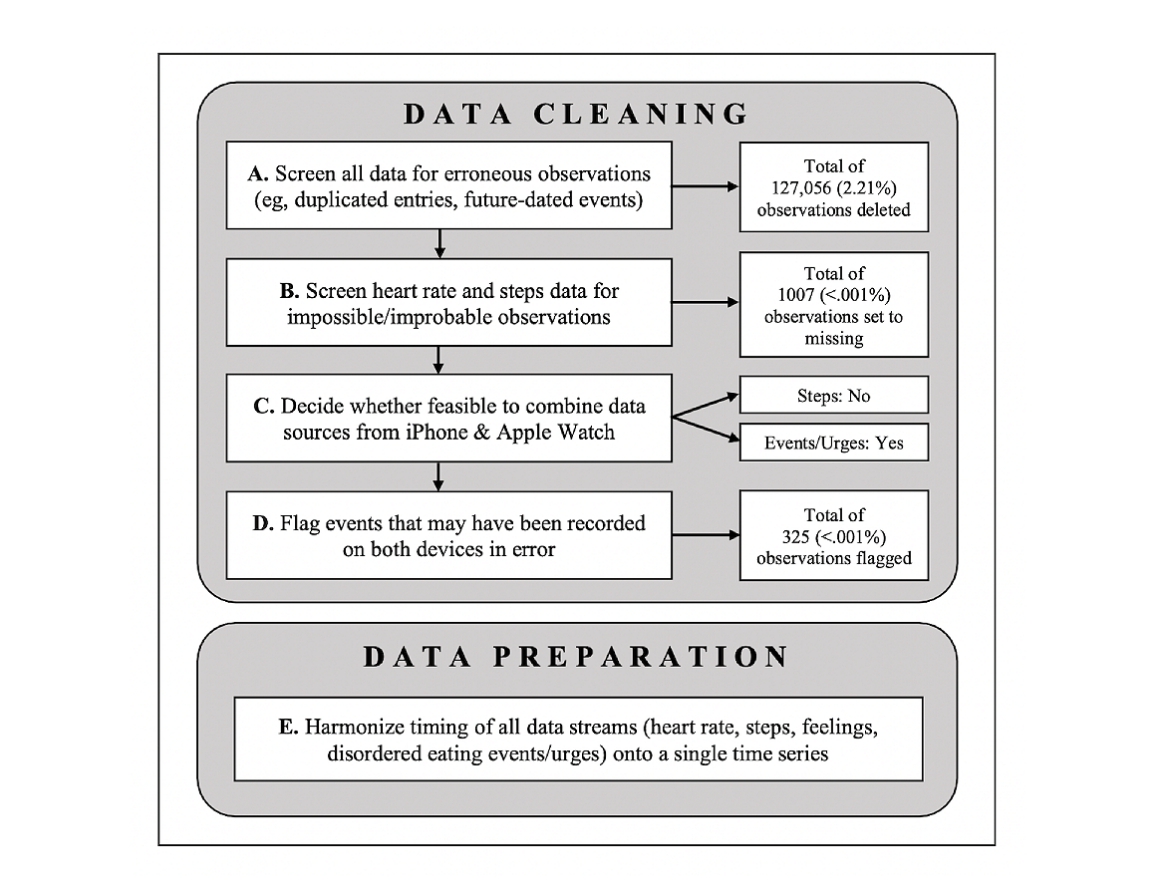
Schematic of all data cleaning and preparation steps.

Figure 1 presents a schematic of all data cleaning and preparation steps. All percentages included in this figure were computed using the total number of precleaning observations available across all data sources. Prior to conducting analyses, all active and passive data were inspected for possible errors in recording or extraction. Details on the numbers and data sources of all raw data, as well as the observations removed from the final dataset prior to analysis, are presented in Table 1 and Table 2.

**Table 1:** Total number of observations across data sources available from iPhone and Apple Watch devices pre–data cleaning.

Data source	Number of observations
Heart rate^a^	3,502,315
Steps	2,118,410
Feelings	85,636
Binge events	7455
Purge events	883
Other eating disorder events^b^	21,431
Binge urges	7460
Purge urges	1219
Other eating disorder urges^c^	8001

^a^Heart rate data collected from Apple Watches only.

^b^Other eating disorder events included restricting, using laxatives, meal skipping, overeating, and compulsively exercising.

^c^Other eating disorder urges include the urge to restrict, urge to use laxatives, urge to meal skip, urge to overeat, and urge to exercise compulsively.

**Table 2:** Summary of observations removed during data cleaning.

Reason for removing a data source^a^		Observations removed, n (%)^b^	Removal method
**Duplicate**			
	Heart rate^c^	82,378 (2.4)	Deleted
	Steps	43,994 (2.1)	Deleted
	Feelings	320 (3.7)	Deleted
	Binge events	40 (0.5)	Deleted
	Purge events	0 (0)	N/A^d^
	Other eating disorder events^e^	72 (0.4)	Deleted
	Binge urges	22 (0.3)	Deleted
	Purge urges	8 (0.7)	Deleted
	Other eating disorder urges^f^	16 (0.2)	Deleted
**Future-dated**			
	Feelings	116 (0.1)	Deleted
	Binge events	23 (0.3)	Deleted
	Purge events	1 (0.1)	Deleted
	Other eating disorder events	28 (0.1)	Deleted
	Binge urges	19 (0.3)	Deleted
	Purge urges	3 (0.3)	Deleted
	Other eating disorder urges	16 (0.2)	Deleted
**Unreliable values**			
	Heart rate	250 (<0.001)	Set to missing
	Steps	757 (<0.001)	Set to missing

^a^Including observations collected from iPhone and Apple Watch devices.

^b^Percentages based on total pre-cleaning number of observations for each data source.

^c^Heart rate data collected from Apple Watches only.

^d^N/A: not applicable.

^e^Other eating disorder events included restricting, using laxatives, meal skipping, overeating, and compulsively exercising.

^f^Other eating disorder urges included the urge to restrict, urge to use laxatives, urge to meal skip, urge to overeat, and urge to compulsively exercise.

#### Duplicate and Future-Dated Data

A total of 126,850 out of 5,752,810 data points, or 2.2% of the total raw data collected, were identified as duplicates and removed from the final data set. A data point was flagged as a duplicate when the timestamp and all associated data characteristics were identical to another observation from the same participant. These values were determined to be device recording errors and were deleted. Similarly, a small percentage of future-dated self-reported events, urges, and feelings were detected (comprising 427 out of 5,752,810 data points, or <0.1% of the total raw data collected). Since future-dating of events was not possible using the phone or watch versions of the Recovery Record app, these observations were attributed to temporary device malfunction and were deleted from the final data set.

#### Device Recording Errors and Unreliable Data

To check for potential recording errors in heart rate values from the Apple Watch (eg, due to the device shifting on the wrist or sweat interfering with the sensor), heart rate data were screened for values outside the typical range for healthy adults (50-150 bpm [20]). We decided that extreme heart rate values immediately preceded and followed by a sudden jump in heart rate (ie, a change greater than 50 bpm) were more likely indicative of device error than meaningful individual differences in cardiovascular fitness or activity and were set to missing. Although 256,590 heart rate observations were flagged as extreme, only 250 of these were classified as likely device recording errors (including 51 observations of 0 bpm) and consequently set to missing. A small number of unreliable or unusable step observations were also detected in the phone and watch data based on the number of steps recorded or the length of the associated recording interval. Step observations that were either impossible (eg, 384 steps recorded in less than one second) or associated with recording intervals that were too long to accurately capture activity dynamics at the desired sensitivity for the current study objectives (ie, greater than 15 minutes), were attributed to device error and set to missing in the final data set. In total, 757 out of 2,118,410 step observations, or less than 0.1% of all step observations recorded, were determined to be unreliable and set to missing.

#### Combining Data Across Devices

With the exception of heart rate, it was possible for participants to generate all forms of mobile data on both the iPhone and Apple Watch devices. Therefore, it was necessary to decide whether data collected from both sources could be combined prior to analysis. First, we inspected the quantity and characteristics of the data obtained from both devices. Table 3 shows descriptive statistics on device utilization across data sources.

**Table 3:** Participant device utilization across data sources.

Data source	Participants who provided the data (N=1019)	Participants who used both devices, n (%)	Participants who used iPhone only, n (%)	Participants who used Apple Watch only, n (%)
Steps	815	771 (94.6)	22 (2.7)	22 (2.7)
Feelings	1013	494 (48.8)	492 (48.6)	27 (2.6)
Binge events	861	283 (32.9)	569 (66.1)	9 (1)
Purge events	119	23 (19.3)	90 (75.6)	6 (5)
Other eating disorder events^a^	911	350 (38.4)	543 (59.6)	18 (2)
Binge urges	816	372 (45.6)	365 (44.7)	79 (9.7)
Purge urges	173	29 (16.8)	135 (78)	9 (5.2)
Other eating disorder urges^b^	722	279 (38.7)	276 (38.2)	167 (23.1)

^a^Other eating disorder events included restricting, using laxatives, meal skipping, overeating, and compulsively exercising.

^b^Other eating disorder urges included the urge to restrict, urge to use laxatives, urge to meal skip, urge to overeat, and urge to compulsively exercise.

##### Passive Data

For steps, 771 out of 815 participants (94.6%) provided data collected from both devices and only a small proportion (22/815, 2.7%) of participants recorded steps only on their iPhone or Apple Watch. Nevertheless, the quantity of step observations recorded on the Apple Watch was much greater than on the iPhone, which is most likely due to participants having the Apple Watch on their person more consistently than the iPhone. As originally planned, and to eliminate the risk of artificially inflating step counts due to simultaneous recording on both devices, we decided to use only step data obtained from the Apple Watch. Because participants did not receive the watch until around 7 days after study enrollment, fewer than 30 days of step data are available for analysis. We opted not to use steps recorded on the iPhone prior to when the Apple Watch was received by participants to limit potential sources of bias from differences in algorithms and sensors between the 2 devices. Step data for the 22 participants who only provided iPhone-generated step counts were therefore excluded, which resulted in a final sample of 793 participants with usable step data for analysis.

##### Active Data

Inspection of active data recorded on the 2 devices revealed that although many participants recorded momentary observations of feelings, disordered eating behaviors, and urges on both the iPhone and Apple Watch versions of the Recovery Record app, most observations came from iPhone entries (Table 3). This was somewhat unexpected, as participants were instructed to use the Apple Watch for these data, and the Apple Watch version of the Recovery Record app was specifically designed to increase ease of active data collection for participants. Since response options for recording eating disorder events, urges, and feelings were consistent across the iPhone and Apple Watch versions of the Recovery Record app, we decided to combine active data collected from both devices prior to analysis. To determine whether participants may have recorded some binge or purge events on both devices by mistake, we flagged any identical events with timestamps that were within 60 minutes of each other. This time interval was chosen based on existing research [21,22] and the clinical expertise of our team, who suggested that it would be unlikely for multiple binge or purge episodes to occur less than 60 minutes apart. A total of 301 out of the 7392 binge events available post–data cleaning (4%) and 24 out of the 882 post–data cleaning purge events (2.7%) were flagged as potential duplicate events to be investigated further using sensitivity analyses.

### Data Preparation

For the analyses proposed for this study (discussed in the Planned Analysis section) it was necessary for us to create corresponding measurements of heart rate and step data that maximized data coverage over time. Although passive heart rate and actigraphy measurements can be assumed to come from continuous data sources, the iPhone and Apple Watch use sampling rates designed to preserve battery power and optimize user experience of other device applications. Furthermore, to maximize user privacy, Apple devices collect location and movement data over irregular time intervals using proprietary sampling algorithms. As a result of these constraints, heart rate and actigraphy data harvested via native application programming interfaces are available as discrete repeated measurements that are unequally and variably spaced across time.

The accuracy of the multivariate analytic approach chosen to address the objectives of the current protocol (also discussed in the Planned Analysis section) depends on observations from data sources being measured simultaneously and at repeated time intervals that capture the underlying continuous dynamics of interest—in this case, the coordination or discoordination between heart rate and steps preceding and following an episode of disordered eating. Therefore, to meet these assumptions, we created a time series for each participant that stretched from their earliest heart rate or step time stamp to their latest heart rate or step time stamp in increments of 1 minute. Time stamps for heart rate and step observations were then truncated to the minute level and heart rate and total step count values were assigned to the time series as follows: Heart rate values were assigned to a 1-minute epoch in the time series corresponding with their time stamps. If, due to truncation of time stamps, multiple heart rate observations were associated with the same minute in the time series, an average of the heart rate observations was computed and assigned to the corresponding minute. For steps, raw step count values were first divided and distributed equally across the number of minutes in their associated time interval. For example, if an observation of 500 steps was associated with a 10-minute time interval, 50 steps would be assigned to each minute within the interval. Step values were then assigned to each corresponding minute in the time series. As with heart rate, whenever multiple step observations were associated with the same minute due to truncation of the raw data to the minute level, an average step value was computed and assigned to the corresponding minute.

### Planned Analysis

Our primary analyses will focus on identifying markers of risk for binge and purge events using a combination of multilevel modeling and mixture modeling techniques. We plan to accomplish this by modeling a person’s current state as a function of their previous state and change in their state over time using values and derivatives of heart rate and step counts. We will run these models both as multilevel models, in which time until the next binge or purge event and time from the previous binge or purge event are included as moderators of these associations, and as mixture models, which take an exploratory approach to identifying different temporal patterns based on distributional properties of the data.

## Results

Data collection, cleaning, and preparation are complete. A total of 1019 participants—including 96 participants from the feasibility pilot—completed the study between August 2017 and May 2021. Summary statistics of participant demographic variables are presented in Table 4. Of the full sample, 859 participants (84.3%) identified their biological sex or gender as female, 148 (14.5%) as male, and 12 participants (1.2%) identified with neither female nor male gender. None of the sample endorsed their race as Pacific Islander or Native Hawaiian, and 61 out of the 1019 participants (6%) identified with multiple racial groups. Of the 1019 participants included in our final sample, 790 (77.5%) provided usable heart rate data, 793 (77.8%) provided usable step observations from the Apple Watch, and 1013 (99.4%) provided usable real time assessments of feelings, disordered eating events, or disordered eating urges on the iPhone or Apple Watch versions of Recovery Record. Summary statistics for the number of days and total number of observations available from each data source after cleaning are presented in Table 5 and Table 6. Analyses for the primary aim will begin in the spring of 2022 and are expected to conclude by the end of 2022.

**Table 4:** Participant demographic data.

Variable		Full sample (N=1019)	Males only (n=148)	Females only (n=859)
Age (years), mean (SD)		29.6 (7.4)	31.7 (7.0)	29.3 (7.4)
**Ethnicity, n (%)**				
	Hispanic	111 (10.9)	15 (10.1)	95 (11.1)
	Non-Hispanic	908 (89.1)	133 (89.9)	764 (88.9)
**Race, n (%)**				
	African American/Black	42 (4.1)	13 (8.8)	28 (3.3)
	American Indian/Alaska Native	6 (<1)	1 (0.7)	5 (0.6)
	Asian	39 (3.8)	11 (7.4)	28 (3.3)
	White	845 (82.9)	114 (77)	723 (84.2)
	More than one race	61 (6)	5 (3.4)	54 (6.3)
	Not reported	26 (2.6)	4 (2.7)	21 (2.5)
**Lifetime diagnosis,^a^ n (%)**				
	Binge eating disorder	787 (77.2)	111 (75)	665 (77.4)
	Bulimia nervosa	759 (74.5)	97 (65.5)	653 (76)
	Binge eating disorder and bulimia nervosa	639 (62.7)	81 (54.7)	549 (63.9)

^a^Indicates that a subject met the diagnostic criteria at some point in their life.

**Table 5:** Summary statistics of the number of days of data available across data sources.

Data source	Mean (SD)	Minimum	Maximum
Heart rate	18 (7)	0	29
Step count	20 (7)	0	30
Feelings, eating disorder events,^a^ or eating disorder urges^b^	23 (8)	0	29
Any	25 (8)	0	30

^a^Eating disorder events included binge eating, purging, restricting, using laxatives, meal skipping, overeating, and compulsively exercising.

^b^Eating disorder urges included the urge to binge, urge to purge, urge to restrict, urge to use laxatives, urge to meal skip, urge to overeat, and urge to compulsively exercise.

**Table 6:** Total number of observations across data sources available from iPhone and Apple Watch devices post–data cleaning.

Data source	Number of observations
Heart rate^a^	3,419,937
Step count^a^	1,635,993
Feelings	85,200
Binge events	7392
Purge events	882
Other eating disorder events^b^	21,332
Binge urges	7419
Purge urges	1208
Other eating disorder urges^c^	7968

^a^Heart rate and step data from Apple Watches only.

^b^Other eating disorder events included restricting, using laxatives, meal skipping, overeating, and compulsively exercising.

^c^Other eating disorder urges included the urge to restrict, urge to use laxatives, urge to meal skip, urge to overeat, and urge to compulsively exercise.

## Discussion

### Contributions

To our knowledge, the digital phenotyping arm of the BEGIN project represents the first study using passive sensor data from personal digital devices to characterize states of increased risk for binge or purge episodes in individuals with BED and BN. By incorporating passive sensor data into mobile health apps, this work has the potential to improve the accessibility, potency, and durability of existing evidence-based treatments for eating disorders. Furthermore, this study lays the foundation for future just-in-time interventions that could utilize real time personal digital device data to alert individuals to impending high-risk states for engaging in eating disorder behaviors and direct them to specific therapeutic intervention strategies when they need them most. This work has the potential to improve therapeutic outcomes in the treatment of BED and BN and can also provide a blueprint for interventions with other health or mental health conditions characterized by discrete events (eg, addictive behaviors such as substance use, smoking, and gambling, and self-injurious or suicidal behaviors).

### Limitations

The current study included only existing iPhone users and relied on the Apple Watch’s native application programming interface for extracting raw passive sensor data. As a result, the data collected may be influenced by patterns of device usage and interaction specific to Apple customers and Apple operating systems. Also, some of our data handling decisions (eg, the definition of unreliable heart rate values) were influenced by the features of our sample and the Apple Watch application programming interface. Therefore, some of these decisions may not be appropriate for studies using more specific samples (eg, athletes) or personal digital devices that provide data at a higher resolution. This study only focused on 2 forms of sensor data (ie, heart rate and step count); however, it is possible to access many other passive data sources from personal digital devices (eg, screen time and call or text logs). This demonstrates that this is an area of research ripe for expansion in the future. Finally, the accuracy and reliability of the participants’ self-reported binge or purge events will influence our ability to capture states of increased risk for these events using sensor data.

### Conclusions

Our study highlights some of the methodological challenges associated with using “out-of-the-box” data from individuals’ existing personal digital devices, and describes a novel approach for addressing these challenges when preparing data for multivariate time series analyses. Given the number of decisions to be made when cleaning and handling personal digital device data, we hope that this protocol will help serve as a guide to other researchers using personal digital devices for digital phenotyping work, while also contributing to greater transparency and reproducibility in this field.

## Appendix

Multimedia Appendix 1 Peer-reviewer report from the Psychosocial Risk and Disease Prevention (PRDP) Study Section - Risk, Prevention and Health Behavior Integrated Review Group - Center for Scientific Review (National Institutes of Health, USA).

## Abbreviations

BED: binge eating disorder
BEGIN: binge eating genetics initiative
BN: bulimia nervosa
PI: Principal Investigator
