# Supplementary material for: Passive Sensor Data for Characterizing States of Increased Risk for Eating Disorder Behaviors in the Digital Phenotyping Arm of the Binge Eating Genetics Initiative: Protocol for an Observational Study
Source: JMIR Res Protoc. 2022 Jun 2;11(6):e38294. doi: 10.2196/38294 (PMC9204566; doi:10.2196/38294)
Supplement: Multimedia Appendix 1 [file resprot_v11i6e38294_app1.pdf]

SUMMARY STATEMENT

(Privileged Communication)

PROGRAM CONTACT:
Mark Chavez
301-443-8942
mchavez1@mail.nih.gov

Release Date: 06/24/2019
Revised Date:

Application Number: 1 R01 MH119084-01A1

Principal Investigators (Listed Alphabetically):

BULIK, CYNTHIA M (Contact)
BUTNER, JONATHAN

Applicant Organization: UNIV OF NORTH CAROLINA CHAPEL HILL

Review Group: PRDP
Psychosocial Risk and Disease Prevention Study Section

Meeting Date: 06/10/2019
Council: OCT 2019
Requested Start: 09/01/2019

RFA/PA: PA19-056
PCC: A2-AIM

Project Title: Predicting Binge and Purge Episodes from Passive and Active Apple Watch Data Using a Dynamical Systems Approach
SRG Action: Impact Score:14 Percentile:4
Next Steps: Visit https://grants.nih.gov/grants/next_steps.htm
Human Subjects: 30-Human subjects involved - Certified, no SRG concerns
Animal Subjects: 10-No live vertebrate animals involved for competing appl.
Gender: 1A-Both genders, scientifically acceptable
Minority: 1U-Minorities and non-minorities, scientifically unacceptable
Age: 7A-Only Adults, scientifically acceptable

Project Year	Direct Costs Requested	Estimated Total Cost
1	499,983	726,545
2	497,870	723,475
3	499,942	726,486
4	499,992	726,558
TOTAL	1,997,787	2,903,064

ADMINISTRATIVE BUDGET NOTE: The budget shown is the requested budget and has not been adjusted to reflect any recommendations made by reviewers. If an award is planned, the costs will be calculated by Institute grants management staff based on the recommendations outlined below in the COMMITTEE BUDGET RECOMMENDATIONS section.

1R01MH119084-01A1 BULIK, CYNTHIA

INCLUSION OF MINORITIES PLAN UNACCEPTABLE

RESUME AND SUMMARY OF DISCUSSION: This application seeks support to conduct a longitudinal investigation of 1000 individuals with bulimia nervosa (BN) or binge-eating disorders (BED) using passive sensor data and active data using Apple Watch to predict BN or BED episodes. This resubmitted work was highly responsive to previous critiques resulting in work that the review panel opines offers exceptionally high impact to address the public health challenge of obesity and in particular to better understand bulimic individuals and to prevent BED. The reviewers again noted many strengths. The exceptional scientific rigor of the premise that triggers can be identified for individuals with BN and BED in this add on study which efficiently leverages the parent study was deemed compelling. The exceptionally novel use of Apple Watch passive and active senor data collection technological assessments to characterize real time to predict BN and BED episodes is convincing; the exceptional investigative team are superb and well supported by an equally exceptional environment; and the outstanding scientific rigor of the approach is strengthened by the self-reports of mood and cognition, Apple Watch assessments, prospective design, large sample size, multi domain data collection and prevention potential for the field of eating disorders. A few minor weaknesses were noted: questions concerning the validity of the respondents actually heeding the trigger alerts, concerns about successful recruitment of sufficient BED and BN individuals and concerns about the potential for missed data preceding a binge episode due to the 5-minute heart rate epoch. In sum, the review panel was very enthusiastic about this high impact work that offers high potential to identify preventive approaches to BN and BED with strengths that far outweigh the few remaining minor weaknesses.

DESCRIPTION (provided by applicant): Bulimia nervosa (BN) and binge eating disorder (BED) are life-interrupting and associated with significant impairment. Via a unique opportunity that allowed us to adapt the widely used cognitive-behavioral based app Recovery Record for use on 1000 Apple Watches, we propose to optimize two domains of data being collected over a 30-day period in 1000 individuals with bulimia nervosa (BN) or binge-eating disorder (BED). This proposal augments a parent study [Binge Eating Genetics INitiative (BEGIN)], supported by NIMH (saliva kits for DNA at no cost). We will collect longitudinal passive sensor data via native applications in the Apple Watch and active data on binge-eating, purging, nutrition, mood, and cognitions using Recovery Record adapted for the Apple Watch. We will combine sensor-based measurements of autonomic nervous system (ANS) activity, actigraphy, and geolocation with active Recovery Record measures to characterize real world conditions under which individuals are more/less likely to binge and/or purge in their daily lives. Applying dynamical systems analytic approaches, both across and within individuals, we will identify stable, low-risk, and high-risk patterns that will enable the prediction of transition to high risk epochs that signal impending binge or purge episodes. Our work will provide an empirical foundation for transcending current cognitive- behavioral therapy approaches that are dependent on self-report (often retrospective) of high-risk states, will enhance the understanding of eating disorders in terms of regulation, and will yield a personalized precision medicine approach to eating disorders treatment. Efficient and reliable quantitative characterization is the essential first step in the development of real-time interventions driven by automated recognition of individualized transitions into high-risk periods for disordered eating behaviors. Our aims are: 1) To predict the occurrence of binge eating and purging episodes in individuals with BN or BED with passive sensor data; 2) To test theoretically-derived regulatory models of binge eating and purging as reflected in differences in temporal patterns; and 3) To refine our capacity to predict high risk states by augmenting passive data with contextual factors collected by Recovery Record. This proposal optimizes the richness and longitudinal structure of the deep phenotypic data collected in BEGIN to lay the foundation for the next translational step in which we will develop personalized just-in-time interventions that can disrupt eating disorders behaviors in real time before they occur.

PUBLIC HEALTH RELEVANCE: We will apply advanced statistical modeling to passive data (via the Apple Watch) and active data (via an eating disorders app Recovery Record) to identify patterns that signal an impending binge or purge episode in 1000 individuals with bulimia nervosa (BN) or binge-eating disorder (BED). By identifying high-risk states, we will lay the foundation for personalized precision treatment by alerting and intervening in individuals with eating disorders before an unhealthy behavior such as binge eating or purging occurs.

CRITIQUE 1

Significance: 2
Investigator(s): 1
Innovation: 1
Approach: 3
Environment: 1

Overall Impact: This is a revised R01 application proposing a highly innovative study aimed at predicting binge eating and purging episodes using active and passive data collection in a sample of 1,000 adults with a diagnosis of bulimia nervosa (BN) or binge-eating disorder (BED). Scientific rigor of prior research is strong: extensive research supports the need to find ways to reduce patient burden arising from self-monitoring and the need to be able to predict high-risk scenarios for the target symptoms. Several weaknesses in articulating scientific rigor have been overcome in the revision. Innovation is high and derives from using technology-supported assessment via a popular i-phone app ("Recovery Records") and Apple watch (using sensory based assessment of physiological measures and geolocation) and the analysis of data using dynamic systems theory methods. The proposed study addresses a problem of high public health significance with a highly innovative research plan, to be conducted by a superb interdisciplinary research team and in an excellent research environment. Scientific rigor is high as reflected in the prospective design, inclusion of a large study sample, a well-justified battery of psychological (active) data combined with the assessment of physiological and other passively collected data, and a clearly described data analysis plan. Several weaknesses remain, including insufficient clarity regarding the composition of the study sample by diagnosis and the absence of compelling data that patients would welcome targeted alerts in high-risk situations. Weaknesses are well off-set by strengths. Expected overall scientific impact is high.

1. Significance:

Strengths

- Binge eating and purging are modifiable health risk behaviors; when unaddressed, they pose considerable risk to health and well-being. Effective interventions are available, yet their reach is less than optimal.
- Patients experience current approaches to self-monitoring as time-consuming and intrusive. Consequently, adherence to this key treatment component can be low. Technology-supported self-monitoring largely overcomes these limitations.
- The proposed study will develop an algorithm to predict high-risk periods or triggers for binge- or purge episodes in individuals with bulimia nervosa (BN) or Binge-Eating Disorder (BED).
- The scientific premise has been strengthened by adding information about the relationship between heart rate and disordered eating.
- The scientific premise also has been strengthened by the addition of a review of the literature on affective changes reported before and after binge eating or purging behavior.

Weaknesses

- It remains unanswered whether individuals would be willing to accept “trigger alerts”—raising questions about the clinical utility and acceptability of the ultimate product of this application. (moderate)

2. Investigator(s):

Strengths

- As was noted in prior critiques, this is a very strong, highly qualified multiple PI team.
- Dr. Cynthia Bulik (MPI), U. of North Carolina, Chapel Hill, is internationally renowned for her eating disorder research which includes studies of the etiology of eating disorders and the development of novel treatments. She has extensive experience in leading highly innovative and productive multidisciplinary research teams.
- Dr. Jonathan Butner (MPI), U. of Utah, is an expert in dynamical systems theory with an excellent record of applying dynamical systems models to health or mental health problems (and other human behavior).
- Co-PI, Dr. Pascal Deboek, a Quantitative Psychologist, adds expertise in repeated intraindividual measurement which he has applied to a wide range of substantive areas including the interplay of stress and affect.
- Dr. Brian Baumcomb, U. of Utah, contributes expertise in intensive measurement and modeling of behavioral and emotional processes associated with psychological dysfunction and maladaptation.
- Co-PI Jena Tregarthen is Co-Founder and Chief Executive Officer of Recovery Record, the technology company that has developed the app to be used in the proposed research. Ms. Tregarthen has co-authored several research papers describing user characteristics and feasibility of the Recovery Record app in the treatment of individuals with an eating disorder.
- Expertise in momentary ecological assessment methods has been added: Dr. Inbal Nahum-Shani brings expertise in novel methodologies and adaptive interventions.

Weaknesses

- None noted.

3. Innovation:

Strengths

- Prior critiques lauded the high level of innovation. These strengths have been preserved.
- The application of dynamical systems theory to the field of eating disorders is highly innovative.
- Combining active and passive data collection of physiological, psychological and geo-location data to identify triggers for binge eating or purging behaviors is innovative.
- Using geolocation data is novel in eating disorder research.

Weaknesses

- None noted.

4. Approach:

Strengths

- A prospective study of 1,000 adults with self-reported BN or BED will collect detailed self-report [active] psychological data on eating behavior, mood, and physiological (heart rate, sleep,

physical activity) and geolocation data [passive], for 30 consecutive days. Self-report data include one-time assessments of eating disorder symptomatology, weekly “fill-in-the-gap” assessments to supplement daily measures, and multiple daily assessments of target behaviors and their circumstances. Passive data will be measured continuously and “epoched” into 5-minute intervals throughout the day. The large sample size, prospective data collection, and rigorous behavioral measures all contribute to high scientific rigor.

- The application now specifies that both event-based (e.g., after binge episodes) and time-based data collection will be used.
- By including of individuals with BN and with BED, the study can address the question of whether signals leading to binge eating are similar in these distinct diagnostic entities.
- The detailed multi-domain data will be integrated using state-of-the-art multilevel modeling and systems continuous time modeling procedures.
- The psychological variables are well-justified based on prior research and cognitive-behavioral theory of eating disorders.
- The fine-grained assessment of physiological measures in participants’ daily life is a strength.
- Collection of geolocation data is a strength.
- The application now provides data that suggest that binge- or purge episodes can be distinguished from regular eating episodes using Loess Vector Plot methodology.
- The revised data analysis plan specifies that the expected reduction over the 30-day observation period in binge or purge episodes will be factored into the analysis plan.
- The accuracy of individualized predictive models will be tested using a split-half design.

Weaknesses

- The application indicates that 400 individuals have enrolled already; it is unclear what proportion of these individuals meet a diagnosis of BN. (minor)
- No pilot data are presented on number of purge episodes or purge days, making it difficult to estimate whether sample size will be adequate for developing the prediction algorithm for purging episodes (minor).
- Information about material benefits to participant Is not specified (e.g., whether participants will be permitted to keep the Apple watch upon completion of the study (minor).
- Validity information regarding the screening questionnaire is based on a sample of individuals with anorexia nervosa; the present study focuses on individuals with BN or BED and, therefore, validity information for BN and BED would be desirable. (minor)

5. Environment:

Strengths

- The UNC Department of Psychiatry, Sheps Center, and University of Utah provide excellent research facilities.

Weaknesses

- None noted.

Study Timeline:

Strengths

- The timeline is appropriate for the scope of this study.

Weaknesses

- None noted

Protections for Human Subjects:

Acceptable Risks and/or Adequate Protections

- Participation in this observational study poses minimal risks. High risk individuals (e.g., suicidality) are screened out.

Data and Safety Monitoring Plan (Applicable for Clinical Trials Only):

Acceptable

- o A Data Safety Monitoring Group will be established; the proposed plan is acceptable.

Inclusion Plans:

- Sex/Gender: Distribution justified scientifically
- Race/Ethnicity: Distribution justified scientifically
- For NIH-Defined Phase III trials, Plans for valid design and analysis:
- Inclusion/Exclusion Based on Age: Distribution justified scientifically
- The study will recruit adults, men and women, ages 18 to 45 years; detailed efforts are described for recruiting individuals representing nimority populations. Altough the application does not specifically justify the upper age limit, exclusion of participants under age 18 is appropriate given that modal onset of BN and BED occurs between age 18 and 25 years. Exclusion of adults over 45 is also scientifically defensible given epidemiological studies showing that a majority of cases with BN or BED are within this age band.

Vertebrate Animals:

Not Applicable (No Vertebrate Animals)

Biohazards:

Not Applicable (No Biohazards)

Resubmission:

- The application is highly responsive to prior critiques. Specifically, scientific premise has been improved by a more complete justification for range of measures to be included for developing the algorithm and by adding discussion of the literature on EMA research in eating disorders. A minor concern about the premise remains, namely whether people would accept "trigger warnings" about impending binge- or purge episodes. Scientific rigor also has been strengthened: the information about assessment has been expanded, clarifying the psychometric properties of the screening instrument; the sample approach has been clarified; new pilot data have been added illustrating that binge eating may be differentiated from non-binge eating episodes; the design now calls for both event-based sampling and random-schedule sampling. The investigative team has been strengthened: expertise in EMA methodology has been added. Effort and contribution of members of the investigative team have been refined and the budget been adjusted or justified accordingly. Human subjects' concerns have been addressed by the inclusion of relevant information. Several minor concerns

remain regarding the scientific approach, but overall the expected impact of this study is deemed high.

Budget and Period of Support:

Recommend as Requested

CRITIQUE 2

Significance: 1
Investigator(s): 1
Innovation: 1
Approach: 3
Environment: 1

Overall Impact: This resubmitted proposal describes a longitudinal study designed to predict binge eating and purging episodes over a 30-day period in a sample of 1000 people with bulimia nervosa (BN) or binge-eating disorder. The investigators propose to combine data collected using a sensor (passive) with a Smartphone app (active) to understand the context of binge-eating behaviors and binge episodes in order to create a repository of factors that may predict a binge episode. Enthusiasm for this study is high, and investigators were highly responsive to previous reviews. The proposal boasts strength in the innovation of the research question and methods, the stellar team, the use of objective measures, and the potential implications of the findings for practice and treatment. Scientific rigor is high; the investigators propose to collect intensive measures of factors that may predict binge episodes. A few issues remain, with respect to missing details on how meals and snacks are defined/timed, and concerns about the potential for missed data preceding a binge episode due to the 5-minute HR epoch. Overall, this study has the potential to make a major impact on the field of disordered eating.

1. Significance:

Strengths

- The ability to predict a binge episode before it occurs (based on contextual and other cues) is significant and may have important disordered eating treatment implications.
- Implications for the potential of methods for providing treatment in a naturalistic environment are compelling. If successful, the resulting programs/technology may reduce barriers to treatment for low-income populations, or those without access to treatment through a clinic.
- The findings will increase current knowledge on antecedents of binge episodes and may move the field forward.

Weaknesses

- None noted.

2. Investigator(s):

Strengths

- Dr. Bulik is an outstanding scientist and leader in the field of eating disorder treatment. Her work has increased our understanding of factors related to disordered eating behaviors, as well as efficacious treatment approaches. Her receipt of ancillary funding from Apple, the National Eating Disorders Association, NARSAD and NIMH adds strength.

- The PI brings together an excellent investigative team with complementary expertise in the areas of dynamical systems modeling (Butner – site PI) and analysis (Deboeck), and statistics (Baucom).
- The addition of Drs. Nahum-Shani and Tregarthen adds much needed expertise in adaptive interventions and Smartphone apps (Dr. Tregarthen led the development of the Smartphone app proposed for use in this study).
- The multiple PI plan is sound.

Weaknesses

- None noted.

3. Innovation:

Strengths

- Combining passive data collection (using sensors on an Apple watch) with active data collection (using a Smartphone app) is novel.
- The ability to understand the context of binge eating, including antecedents and outcomes is novel.

Weaknesses

- None noted.

4. Approach:

Strengths

- Capitalizing on the use of an existing study of eating disorders (BEGIN).
- Participants are recruited from across the country, which may increase the investigators' ability to generalize findings to adults with BN and BE in the U.S.
- Preliminary findings provide evidence of feasibility and promise for the proposed study.
- The dynamical systems modeling approach and analysis.
- Providing an extension of clinic-based treatment with treatment in a naturalistic environment.
- Revisions provide much-needed details on study methods and procedures.

Weaknesses

- Regarding daily mood and meal records, investigators state that participants receive a notification corresponding to meal and snack times. How are these times determined? Based on user-defined schedules? (moderate)
- Is it possible that important information will be missed due to the use of 5-minute epochs for heart rate data? For example, the Apple Watch can be set to provide an alert when heart rate changes markedly or is outside of a specified age range. Is it possible that changes in heart rate may be missed during a period when other contextual binge-eating triggers are present? (moderate-major)

5. Environment:

Strengths

- The facilities, resources and research environment at the University of North Carolina, the Karolinska Institute, and the University of Utah will provide excellent support for this project.

Weaknesses

- None noted.

Study Timeline:

Strengths

- Sufficient time is built in for development of infrastructure, piloting, recruitment, data collection and data management.

Weaknesses

- None noted.

Protections for Human Subjects:

Acceptable Risks and/or Adequate Protections

- Procedures to minimize risk and protect confidentiality were adequately outlined.

Data and Safety Monitoring Plan (Applicable for Clinical Trials Only):

Acceptable

- A DSMP and Data Monitoring Group was outlined with oversight by the study team.

Inclusion Plans:

- Sex/Gender: Distribution justified scientifically
- Race/Ethnicity: Distribution justified scientifically
- For NIH-Defined Phase III trials, Plans for valid design and analysis: Not applicable
- Inclusion/Exclusion Based on Age: Distribution justified scientifically
- Participants will include 600 male and female adults (expected to be predominantly White and predominantly female). These numbers reflect the current demographic of Recovery Record users.

Vertebrate Animals:

Not Applicable (No Vertebrate Animals)

Biohazards:

Not Applicable (No Biohazards)

Resubmission:

- The investigators were highly responsive to the previous reviews. Most, if not all concerns were addressed, and the study methods and procedures were revised accordingly.

Applications from Foreign Organizations:

Justified

- The PI has a joint appointment at University of North Carolina and the Karolinska Institute in Sweden.

Resource Sharing Plans:

Acceptable

Budget and Period of Support:

Recommend as Requested

CRITIQUE 3

Significance: 1
Investigator(s): 1
Innovation: 1
Approach: 1
Environment: 1

Overall Impact: The overall goal of this application is to be able to predict binge eating and purging episodes using passive and active data sources collected from a large sample (~1000) individuals suffering from Bulimia Nervosa (BN) or Binge Eating Disorder (BED). BN and BED are chronic conditions that don't always respond well to traditional treatments. Moreover, the gold-standard interventions are costly, time consuming and not widely available. Understanding the antecedents of binge episodes using widely available technology will help to craft new, precision treatments for both disorders. Specifically, the aims are to predict the occurrence of binge eating and purging episodes using passive sensor data; test regulatory models of binge eating and purging behaviors based on temporal patterns, and; augment passive data collected with active data and contextual factors collected with a previously developed app called Recovery Record. The scientific premise is strong and has been enhanced since the last submission by the addition of information about heart rate monitoring and binge episodes. The investigative team is excellent and now includes consultation with a JITAI expert and the methodology is rigorous. The application is also very innovative. While the overall impact on BN and BED may be moderate, the approach that will be tested in this application can inform the development of other e and mHealth applications for various chronic diseases which increases the overall impact of the project.

1. Significance:

Strengths

- BN and BED are serious health disorders that are associated with a number of medical and psychological problems.
- Understanding the predictors of binge episodes can open up a new approach to treatment.
- The use of scalable technology will allow for broad dissemination

Weaknesses

- None noted.

2. Investigator(s):

Strengths

- The investigative team is excellent and includes expertise in eating disorders, JITAI interventions, app development and statistical modeling. The team is also strengthened by the inclusion of data management and data safety experts.

Weaknesses

- None noted.

3. Innovation:

Strengths

- The use of Apple Watches and an iPhone app is innovative especially as they are allowing for the combination of passive and active data collection.
- The use of complex or dynamic systems theory to untangle the predictors of binge eating episodes is novel.

Weaknesses

- None noted.

4. Approach:

Strengths

- The use of an existing app (Recovery Record) and an existing study (BEGIN) to leverage resources.
- The preliminary data on retention and adherence are impressive.
- The “big data” approach to handling the large volume of data expected represents significant rigor for the application.
- The addition of a modeling and “goodness of fit” approach that uses half of each subjects’ data to develop a model and the other half to confirm the model.
- The decision to evaluate group data as well as individual data to see if anything can be gained with an individual approach.
- The additional explanation of the projected response to the app in terms of binge episodes and whether they will be affected is reassuring.

Weaknesses

- None noted.

5. Environment:

Strengths

- The environment is excellent at each institution.

Weaknesses

- None noted.

Study Timeline:

Strengths

- The timeline appears appropriate for the proposed recruitment and data collection schedule.

Weaknesses

- None noted.

Protections for Human Subjects:

Acceptable Risks and/or Adequate Protections

- The risks to subjects are adequately addressed and a suicide protocol has been added.

Data and Safety Monitoring Plan (Applicable for Clinical Trials Only):

Not Applicable (No Clinical Trials)

Inclusion Plans:

- Sex/Gender: Distribution justified scientifically
- Race/Ethnicity: Distribution justified scientifically
- For NIH-Defined Phase III trials, Plans for valid design and analysis: Not applicable
- Inclusion/Exclusion Based on Age: Distribution justified scientifically
- The application seeks to enroll both men and women, adults over 18 and approximately 23% minority participants.

Vertebrate Animals:

Not Applicable (No Vertebrate Animals)

Biohazards:

Not Applicable (No Biohazards)

Resubmission:

- This application is a resubmission and all major concerns have been addressed.

Resource Sharing Plans:

Acceptable

Budget and Period of Support:

Recommend as Requested

THE FOLLOWING SECTIONS WERE PREPARED BY THE SCIENTIFIC REVIEW OFFICER TO SUMMARIZE THE OUTCOME OF DISCUSSIONS OF THE REVIEW COMMITTEE, OR REVIEWERS' WRITTEN CRITIQUES, ON THE FOLLOWING ISSUES:

PROTECTION OF HUMAN SUBJECTS: ACCEPTABLE

INCLUSION OF WOMEN PLAN: ACCEPTABLE

INCLUSION OF MINORITIES PLAN: UNACCEPTABLE. Reviewers recommend oversampling minorities.

INCLUSION OF CHILDREN PLAN: ACCEPTABLE

COMMITTEE BUDGET RECOMMENDATIONS: The budget was recommended as requested.

Footnotes for 1 R01 MH119084-01A1; PI Name: BULIK, CYNTHIA M

NIH has modified its policy regarding the receipt of resubmissions (amended applications). See Guide Notice NOT-OD-14-074 at http://grants.nih.gov/grants/guide/notice-files/NOT-OD-14-074.html. The impact/priority score is calculated after discussion of an application by averaging the overall scores (1-9) given by all voting reviewers on the committee and multiplying by 10. The criterion scores are submitted prior to the meeting by the individual reviewers assigned to an application, and are not discussed specifically at the review meeting or calculated into the overall impact score. Some applications also receive a percentile ranking. For details on the review process, see http://grants.nih.gov/grants/peer_review_process.htm#scoring.

MEETING ROSTER

Psychosocial Risk and Disease Prevention Study Section
Risk, Prevention and Health Behavior Integrated Review Group
CENTER FOR SCIENTIFIC REVIEW
PRDP
06/10/2019 - 06/11/2019

Notice of NIH Policy to All Applicants: Meeting rosters are provided for information purposes only. Applicant investigators and institutional officials must not communicate directly with study section members about an application before or after the review. Failure to observe this policy will create a serious breach of integrity in the peer review process, and may lead to actions outlined in NOT-OD-14-073 at https://grants.nih.gov/grants/guide/notice-files/NOT-OD-14-073.html and NOT-OD-15-106 at https://grants.nih.gov/grants/guide/notice-files/NOT-OD-15-106.html, including removal of the application from immediate review.

CHAIRPERSON(S)

JAKICIC, JOHN M, PHD
PROFESSOR AND CHAIR
DEPARTMENT OF HEALTH
AND PHYSICAL ACTIVITY
UNIVERSITY OF PITTSBURGH
PITTSBURGH, PA 15261

MEMBERS

APOVIAN, CAROLINE M, MD *
PROFESSOR
DEPARTMENT OF MEDICINE AND PEDIATRICS
NUTRITION AND WEIGHT MANAGEMENT CENTER
BOSTON UNIVERSITY SCHOOL OF MEDICINE
BOSTON, MA 02118

APPELHANS, BRADLEY M, PHD
ASSOCIATE PROFESSOR
DEPARTMENT OF PREVENTIVE MEDICINE
RUSH UNIVERSITY MEDICAL CENTER
CHICAGO, IL 60612

BERRY, DIANE C, PHD *
PROFESSOR
SCHOOL OF NURSING
UNIVERSITY OF NORTH CAROLINA AT CHAPEL HILL
CHAPEL HILL, NC 27599

BUMAN, MATTHEW P, PHD
ASSOCIATE PROFESSOR
COLLEGE OF HEALTH SOLUTIONS
ARIZONA STATE UNIVERSITY
PHOENIX, AZ 85004

DEMARK-WAHNEFRIED, WENDY, PHD
PROFESSOR
DEPARTMENT OF NUTRITION SCIENCES
UNIVERSITY OF ALABAMA AT BIRMINGHAM
BIRMINGHAM , AL 35294

DUTTON, GARETH R, PHD
PROFESSOR
DIVISION OF PREVENTIVE MEDICINE
DEPARTMENT OF MEDICINE
UNIVERSITY OF ALABAMA AT BIRMINGHAM
BIRMINGHAM, AL 35205

FITZPATRICK, STEPHANIE LENAY, PHD
INVESTIGATOR
CENTER FOR HEALTH RESEARCH
KAISER FOUNDATION RESEARCH INSTITUTE
PORTLAND, OR 97227

FRANCIS, LORI ANNE, PHD
ASSOCIATE PROFESSOR
DEPARTMENT OF BIOBEHAVIORAL HEALTH
PENNSYLVANIA STATE UNIVERSITY
UNIVERSITY PARK, PA 16802

FUEMMELER, BERNARD F, PHD
PROFESSOR
DEPARTMENT OF HEALTH BEHAVIOR AND POLICY
MASSEY CANCER CENTER
VIRGINIA COMMONWEALTH UNIVERSITY
RICHMOND, VA 23298

HARVEY, JEAN R, PHD
PROFESSOR AND CHAIR
DEPARTMENT OF NUTRITION AND FOOD SCIENCES
UNIVERSITY OF VERMONT
BURLINGTON, VT 05405

LEWIS, BETH A, PHD *
PROFESSOR AND DIRECTOR
SCHOOL OF KINESIOLOGY
UNIVERSITY OF MINNESOTA
MINNEAPOLIS, MN 55455

LEWIS, MEGAN A, PHD *
SENIOR SCIENTIST
CENTER FOR COMMUNICATION SCIENCE
RTI INTERNATIONAL
RESEARCH TRIANGLE PARK, NC 27709

LUMENG, JULIE C, MD
PROFESSOR
DEPARTMENT OF PEDIATRICS
UNIVERSITY OF MICHIGAN, ANN ARBOR
ANN ARBOR, MI 48109

MAY, LINDA ELIZABETH, PHD *
ASSOCIATE PROFESSOR
EAST CAROLINA UNIVERSITY
GREENVILLE, NC 27834

MENDOZA, JASON A, MD
ASSOCIATE PROFESSOR
DEPARTMENT OF PEDIATRICS
SEATTLE CHILDREN'S HOSPITAL
UNIVERSITY OF WASHINGTON SCHOOL OF MEDICINE
SEATTLE, WA 98145

MUCCI, LORELEI, SCD *
ASSOCIATE PROFESSOR
DEPARTMENT OF EPIDEMIOLOGY
HARVARD SCHOOL OF PUBLIC HEALTH
HARVARD UNIVERSITY
BOSTON, MA 02115

NAPOLITANO, MELISSA A, PHD
PROFESSOR
DEPARTMENTS OF PREVENTION
AND COMMUNITY HEALTH, EXERCISE
AND NUTRITION SCIENCES
GEORGE WASHINGTON UNIVERSITY
WASHINGTON, DC 20052

NEELON, SARA ELIZABETH, PHD
ASSOCIATE PROFESSOR
DEPARTMENT OF HEALTH
BEHAVIOR AND SOCIETY
JOHNS HOPKINS SCHOOL OF PUBLIC HEALTH
BALTIMORE , MD 21205

NOCK, NORA L, PHD
ASSOCIATE PROFESSOR
DEPARTMENT OF EPIDEMIOLOGY AND BIOSTATISTICS
CASE WESTERN COMPREHENSIVE CANCER CENTER
CASE WESTERN RESERVE UNIVERSITY
CLEVELAND , OH 44106

RAYNOR, HOLLIE A, PHD
PROFESSOR
DEPARTMENT OF NUTRITION
UNIVERSITY OF TENNESSEE
KNOXVILLE, TN 37996

RHEE, KYUNG E, MD
ASSOCIATE PROFESSOR
DEPARTMENT OF PEDIATRICS
UNIVERSITY OF CALIFORNIA, SAN DIEGO
LA JOLLA, CA 92093

ROSAS, LISA GOLDMAN, PHD *
ASSISTANT PROFESSOR
DIVISIONS OF EPIDEMIOLOGY AND PRIMARY CARE
AND POPULATION HEALTH
STANFORD UNIVERSITY SCHOOL OF MEDICINE
PALO ALTO, CA 94305

SCHNEIDER, MARGARET L, PHD
PROFESSOR
DEPARTMENTS OF URBAN PLANNING AND PUBLIC POLICY
INSTITUTE FOR CLINICAL AND TRANSLATIONAL SCIENCE
UNIVERSITY OF CALIFORNIA, IRVINE
IRVINE, CA 92617

SEVICK, MARY A, SCD
PROFESSOR
DEPARTMENT OF POPULATION HEALTH
SCHOOL OF MEDICINE
NEW YORK UNIVERSITY
NEW YORK, NY 10016

STAPLETON, JEROD LYNN, PHD *
ASSOCIATE PROFESSOR
DEPARTMENT OF MEDICINE RUTGERS
THE STATE UNIVERSITY OF NEW JERSEY
NEW BRUNSWICK, NJ 08903

WARING, MOLLY E., PHD *
ASSISTANT PROFESSOR
DEPARTMENT OF ALLIED HEALTH SCIENCES
COLLEGE OF AGRICULTURE HEALTH AND NATURAL
RESOURES
UNIVERSITY OF CONNECTICUT
STORRS, CT 06269

WEISSMAN, RUTH STRIEGEL, PHD *
PROFESSOR
DEPARTMENT OF PSYCHOLOGY
WESLEYAN UNIVERSITY
MIDDLETOWN, CT 06459

WILLIAMS, DAVID M, PHD
ASSOCIATE PROFESSOR
DEPARTMENT OF BEHAVIORAL AND SOCIAL SCIENCES
CENTER FOR HEALTH EQUITY RESEARCH
BROWN UNIVERSITY SCHOOL OF PUBLIC HEALTH
PROVIDENCE, RI 02912

SCIENTIFIC REVIEW OFFICER

FITZSIMMONS, STACEY, PHD
SCIENTIFIC REVIEW OFFICER
CENTER FOR SCIENTIFIC REVIEW
NATIONAL INSTITUTES OF HEALTH
BETHESDA, MD 20892

EXTRAMURAL SUPPORT ASSISTANT

FAYEMIWO, TOLU
EXTRAMURAL SUPPORT ASSISTANT
CENTER FOR SCIENTIFIC REVIIEW
NATIONAL INSTITUTES OF HEALTH
BETHESDA, MD 20892

* Temporary Member. For grant applications, temporary members may participate in the entire meeting or may review only selected applications as needed.

Consultants are required to absent themselves from the room during the review of any application if their presence would constitute or appear to constitute a conflict of interest.
